# Mathematical Analysis of Viral Replication Dynamics and Antiviral Treatment Strategies: From Basic Models to Age-Based Multi-Scale Modeling

**DOI:** 10.3389/fmicb.2018.01546

**Published:** 2018-07-11

**Authors:** Carolin Zitzmann, Lars Kaderali

**Affiliations:** Institute of Bioinformatics and Center for Functional Genomics of Microbes, University Medicine Greifswald, Greifswald, Germany

**Keywords:** mathematical modeling, viral kinetics, viral replication, human immunodeficiency virus, Hepatitis C virus, Influenza A virus, antiviral therapy, immune response

## Abstract

Viral infectious diseases are a global health concern, as is evident by recent outbreaks of the middle east respiratory syndrome, Ebola virus disease, and re-emerging zika, dengue, and chikungunya fevers. Viral epidemics are a socio-economic burden that causes short- and long-term costs for disease diagnosis and treatment as well as a loss in productivity by absenteeism. These outbreaks and their socio-economic costs underline the necessity for a precise analysis of virus-host interactions, which would help to understand disease mechanisms and to develop therapeutic interventions. The combination of quantitative measurements and dynamic mathematical modeling has increased our understanding of the within-host infection dynamics and has led to important insights into viral pathogenesis, transmission, and disease progression. Furthermore, virus-host models helped to identify drug targets, to predict the treatment duration to achieve cure, and to reduce treatment costs. In this article, we review important achievements made by mathematical modeling of viral kinetics on the extracellular, intracellular, and multi-scale level for Human Immunodeficiency Virus, Hepatitis C Virus, Influenza A Virus, Ebola Virus, Dengue Virus, and Zika Virus. Herein, we focus on basic mathematical models on the population scale (so-called target cell-limited models), detailed models regarding the most important steps in the viral life cycle, and the combination of both. For this purpose, we review how mathematical modeling of viral dynamics helped to understand the virus-host interactions and disease progression or clearance. Additionally, we review different types and effects of therapeutic strategies and how mathematical modeling has been used to predict new treatment regimens.

## Introduction

Viruses are small obligate intracellular parasites that are unable to reproduce independent of their host. Outbreaks of infectious viral diseases are a major global health concern, a circumstance that is evident by recent large epidemics of influenza, zika fever, Ebola virus disease, and the Middle East Respiratory Syndrome (MERS). According to the United Nations, the recent zika outbreak caused socio-economic costs of approximately US$7-18 billion in Latin America and the Caribbean from 2015 to 2017 (United Nations, 2017). A recent study estimated the socio-economic costs for symptomatic dengue cases (58.40 million) with US$8.9 billion in 141 countries in 2013 (Shepard et al., 2016). This number is expected to rise further in the coming years. Factors such as climate change and increasing air travel are furthermore increasing the risk of global pandemic infections; examples are recent global influenza outbreaks as much as the emergence of tropical infections such as Dengue Virus infections in previously unaffected regions in the United States and Europe (Mackey et al., 2014). To control this global threat, novel therapeutic and antiviral treatment approaches are urgently needed. To amplify the development of such novel drugs and to optimize treatment strategies, a comprehensive understanding of the viral infection dynamics, their parasitic interaction with their host, as well as host defense strategies against the invader are of major importance. In recent years, targeting viral agents that are essential for the viral replication has proven highly effective (Asselah et al., 2016). However, the emergence of resistance against these direct acting antiviral compounds leads more and more to treatment failure and multi-drug resistant viral strains (Poveda et al., 2014). In order to circumvent drug-resistance, novel antiviral strategies focus on the host by supporting the immune response or targeting host factors required for the viral life cycle. The advantage of these methods are higher barriers for the development of resistance and novel opportunity of broad-spectrum antivirals (Zeisel et al., 2013).

Mathematical modeling has proven to be a powerful tool to study viral pathogenesis and has yielded insights into the intracellular viral infection dynamics, the effect of the immune system, the evaluation of treatment strategies, and the development of drug resistance (Bonhoeffer et al., 1997; Perelson, 2002; Rong and Perelson, 2009; Perelson and Ribeiro, 2013; Boianelli et al., 2015; Perelson and Guedj, 2015; Ciupe and Heffernan, 2017). Modeling can deepen our understanding on different scales: From the molecular scale of intracellular virus-host interactions, extracellular cell-to-cell infection at the population scale, to virus spread within organs or whole organisms (Kumberger et al., 2016). In order to quantitatively study the viral growth at a molecular level and to investigate host requirements and limitations, first intracellular models have been developed for bacteriophages (Buchholtz and Schneider, 1987; Eigen et al., 1991; Endy et al., 1997), Baculovirus (Dee and Shuler, 1997), and Semliki Forest Virus (Dee et al., 1995). By studying cell-to-cell infection, early models for Human Immunodeficiency Virus (HIV) (Ho et al., 1995; Wei et al., 1995; Perelson et al., 1996, 1997; Stafford et al., 2000) provided insights into the pathogenesis, treatment strategies, and virus control by the immune system.

On the population scale, the target cell-limited model (Nowak and Bangham, 1996; Nowak et al., 1996; Bonhoeffer et al., 1997; Perelson, 2002; Wodarz and Nowak, 2002) has been extensively used to investigate the virus-host interaction of HIV, Hepatitis C Virus (HCV), and Influenza A Virus (IAV), which will be explained in this review in more detail. Furthermore, we describe the latest achievements made by modeling the dynamics of Ebola Virus (EBOV), Dengue Virus (DENV), and Zika Virus (ZIKV) that caused the most recent viral outbreaks. In addition, we give an introduction into the target cell-limited model with its extensions and applications to investigate the effects of direct antiviral therapy and immune response and highlight the most important achievements made by viral modeling of the intracellular, extracellular and the integration of both, the multi-scale level.

## The target cell-limited model and its extensions

### Target cell-limited model

**Figure 1:**
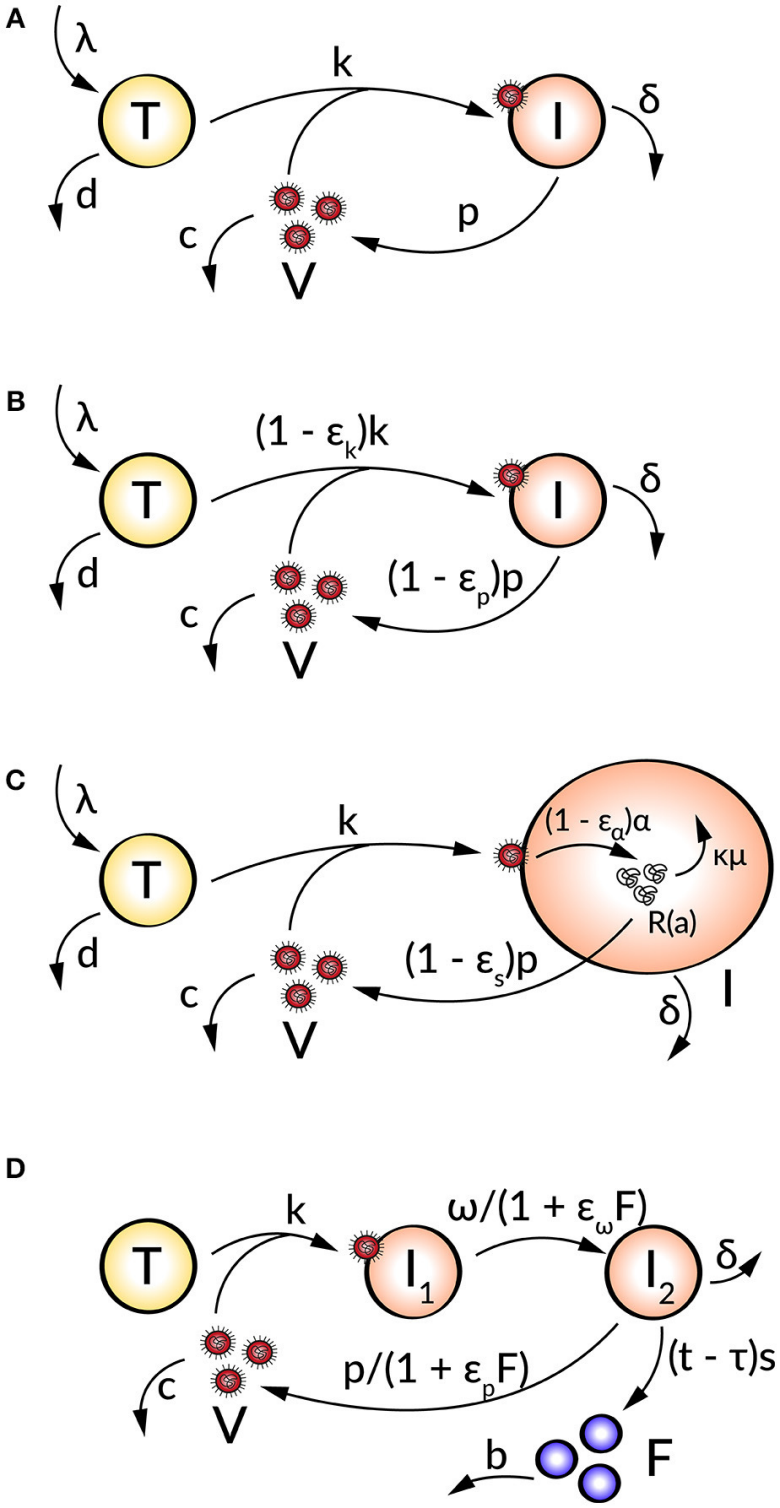
Schematic illustration of **(A)** the target cell-limited model, **(B)** the target cell-limited model extended by antiviral inhibition, **(C)** the age-based multi-scale model, and **(D)** the target cell-limited model extended by the innate immune response.

The first mathematical models described the HIV progression by neglecting intracellular processes and taking only the key players of the virus-host interaction into account (Perelson et al., 1993, 1996, 1997; Ho et al., 1995; Bonhoeffer et al., 1997). The target cell-limited model (Figure 1A) includes three species: uninfected susceptible target cells (*T*), infected virus-producing cells (*I*), and the virus load (*V*) and is formulated by the following system of nonlinear ordinary differential equations (ODEs):

(1) $$\begin{array}{l} \frac{dT}{dt}=\lambda -dT-kVT,\\ \;\frac{dI}{dt}=kVT-\delta I,\\ \frac{dV}{dt}=pI-cV. \end{array}$$

Uninfected target cells (*T*) are produced at a constant rate λ and die at rate *d*, corresponding to a target cell half-life of $t_{T_{1/2}}=\frac{\ln\left(2\right)}{d}$. By the interaction of virus (*V*) with uninfected target cells (*T*) at a constant infectivity rate *k*, the target cells become infected cells (*I*), which in turn produce infectious virus (*V*) with production rate *p*. Due to viral cytopathicity, immune elimination and/or apoptosis, infected cells (*I*) die at a rate δ [resulting in an infected cell half-life $t_{I_{1/2}}=\frac{\ln\left(2\right)}{\delta }$]. Virus is cleared at rate *c* from the cells [virion half-life $t_{V_{1/2}}=\frac{\ln\left(2\right)}{c}$] per virion by mechanisms such as immune elimination (Nowak and Bangham, 1996; Nowak et al., 1996; Bonhoeffer et al., 1997; Perelson, 2002; Wodarz and Nowak, 2002).

With average lifetimes of 1/*d*, 1/δ, and 1/*c* for uninfected target cells, infected cells, and virus, respectively, the total number of virus particles *N* produced by one infected cell during its lifetime is calculated by *p*/δ. Therefore, the production rate *p* of one infected cell is *p* = *Nδ*. Without a viral infection (*I* = 0 and *V* = 0), target cells are in equilibrium with λ/*d* (Nowak and May, 2001; Perelson, 2002; Wodarz and Nowak, 2002).

The ability of a virus to develop an infection or to be cleared is given by the basic reproductive ratio $R_{0}=\frac{\lambda kp}{d\delta c}.\;R_{0}$ represents the number of productively infected cells newly generated by one productively infected cell. With *R*_0_>1 the infection grows due to an increase in virus-producing infected cells while *R*_0_ < 1 refers to a decrease in productively infected cells and viral clearance (Nowak and May, 2001).

### Target cell-limited model and antiviral therapy

To analyze the effect of antiviral drugs that either block infection (ε_*k*_) and/or production of viral particles (ε_*p*_), the target cell-limited model is modified as follows (Figure 1B):

(2) $$\begin{array}{l} \frac{dT}{dt}=\lambda -dT-\left(1-\varepsilon_{k}\right)kVT,\\ \;\frac{dI}{dt}=\left(1-\varepsilon_{k}\right)kVT-\delta I,\\ \frac{dV}{dt}=\left(1-\varepsilon_{p}\right)pI-cV, \end{array}$$

with 0 ≤ ε_*k, p*_ ≤ 1 (Neumann, 1998). Here, ε_*k, p*_ = 0 describes no drug effect while ε_*k, p*_ = 1 refers to the case of a 100% effective treatment—a perfect drug. Note that before treatment ε_*k, p*_ = 0. In simulating treatment, one assumes that the system is in steady state at treatment initiation, at which point the infection and/or production rates are modified depending on the type of antiviral drug used (ε_*k*_ > 0 and/or ε_*p*_ > 0). The overall drug efficacy ε_*tot*_ may be calculated as ε_*tot*_ = 1−(1 − ε_*k*_)(1 − ε_*p*_), while the critical drug efficacy ε_*c*_ is given by $\varepsilon_{c}=1-\frac{d\delta c}{\lambda kp}$ and determines the transition from viral eradication to viral persistence. A successful drug therapy would clear the virus with ε_*tot*_ > ε_*c*_ while the infection becomes chronic when ε_*tot*_ < ε_*c*_ (Dahari et al., 2007a).

The relationship between a certain drug dose and the resulting response can be integrated into the target cell-limited model by the simple time-dependent pharmacodynamic equation

(3) $$\varepsilon \left(t\right)=\frac{\varepsilon_{max}\cdot C{\left(t\right)}^{n}}{EC_{50}{}^{n}+C{\left(t\right)}^{n}},$$

where ε_*max*_ describes the maximum of the drug effect, *EC*_50_ the drug concentration with 50% efficacy, and *C*(*t*) the drug concentration or dose applied (Holford and Sheiner, 1982). Depending on the shape and steepness of the underlying drug effect, the Hill coefficient *n* describes either a sigmoidal curve for *n* > 1 or a hyperbolic curve otherwise. By substituting *C*(*t*) by *C*(*t* − τ), a pharmacodynamic delay τ for the drug effect can be taken into account for *t* > τ (Holford and Sheiner, 1982; Guedj et al., 2010; Canini and Perelson, 2014).

### Age-based multi-scale model for direct acting antivirals

Age-based multi-scale models have been used in order to study the modes of action of antivirals within a virus-infected cell (Nelson et al., 2004; Guedj et al., 2013; Heldt et al., 2013; Clausznitzer et al., 2015). To include the effect of direct acting antivirals (DAAs), the target cell-limited model can be further extended by more detailed intracellular processes of the viral life cycle (Figure 1C). These multi-scale models that take the age of infected cells into account allow a biologically more realistic representation of intracellular processes with age-dependent reaction rates (Quintela et al., 2017). The target cell-limited model coupled to intracellular processes and an age-dependency is formulated as follows:

(4) $$\begin{array}{l} \;\frac{dT}{dt}=\lambda -dT-kTV,\\ \;\frac{\partial I}{\partial a}+\frac{\partial I}{\partial t}=\delta I\left(a,t\right),\\ \frac{\partial R}{\partial a}+\frac{\partial R}{\partial t}=\left(1-\varepsilon_{\alpha }\right)\alpha -\kappa \mu R-\left(1-\varepsilon_{s}\right)\rho R,\\ \;\frac{dV}{dt}=\left(1-\varepsilon_{s}\right)\rho \int_{0}^{\infty }R\left(a,t\right)I\left(a,t\right)da-cV, \end{array}$$

with boundary and initial conditions *I*(0, *t*) = *kVT*, *I*(*a*, 0) = *I*_0_(*a*), *R*(0, *a*) = 1, and *R*(*a*, 0) = *R*_0_(*a*) (Guedj et al., 2013). Here, the intracellular viral genome (*R*) is produced at constant rate α and degraded at constant rate μ. The progeny virions are assembled and secreted at constant rate ρ. The drug effects regard intracellular processes or the viral genome replication: blocking viral RNA production ε_α_ and virion assembly/secretion ε_*s*_, as well as increasing viral RNA degradation κ for κ > 1. Note that the intracellular viral genome [*R*(*a*)] and infected cells [*I*(*a*)] are dependent on the age *a* of the cell, measured as time elapsed since infection, and viral RNA levels increase with the age of the infected cell (Guedj et al., 2013; Canini and Perelson, 2014; Perelson and Guedj, 2015).

### Extended target cell-limited model by the immune response

The innate and adaptive immune response provide various mechanisms in fighting a viral infection. The innate immune response (IIR) represents the first line of defense that recognizes the virus and triggers the adaptive immune response (AIR) (Braciale et al., 2013; Iwasaki and Medzhitov, 2013). In order to study the effect of the immune response on the viral dynamics, mathematical models incorporate key players of the immune response which inhibit processes in the viral life cycle. A further modification of the target cell-limited model has been developed to take the effect of the cell's IIR into account (Figure 1D). This is done by including the effect of interferon (IFN) into the model:

(5) $$\begin{array}{l} \frac{dT}{dt}=-kTV,\\ \frac{dI_{1}}{dt}=kTV-\frac{\omega }{1+\varepsilon_{\omega }F}I_{1},\\ \frac{dI_{2}}{dt}=\frac{\omega }{1+\varepsilon_{\omega }F}I_{1}-\delta I_{2}-sI_{2}\left(t-\tau \right)F,\\ \frac{dV}{dt}=\frac{p}{1+\varepsilon_{p}F}I_{2}-cV,\\ \frac{dF}{dt}=sI_{2}\left(t-\tau \right)-bF. \end{array}$$

Herein, two populations of infected cells *I*_1_ and *I*_2_ describe a time delay. Infected but not yet virus producing cells (*I*_1_) in the eclipse phase become productively virus producing cells (*I*_2_) with average transition time $1/\frac{\omega }{1+\varepsilon_{\omega }F}$. Note that *I*_1_ are not dying before the transition into *I*_2_. Following a time delay τ for the IIR, IFN (*F*) is secreted by *I*_2_ at constant rate *s* and degrades at constant rate *b*. The effect of IFN has been modeled by decreasing the transition rate ω and/or the virus production rate *p* and effectiveness ε_ω_ and ε_*p*_ (Baccam et al., 2006).

Moreover, the effect of the IIR and the AIR can be coupled with the target cell-limited model by simple assumptions:

(6) $$\begin{array}{l} \;\frac{dT}{dt}=rD-kTV,\\ \;\frac{dI_{1}}{dt}=kTV-\omega I_{1},\\ \;\frac{dI_{2}}{dt}=\omega I_{1}-\delta I_{2},\\ \;\frac{dD}{dt}=\delta I_{2}-rD,\\ \;\frac{dV}{dt}=\frac{p}{1+\varepsilon_{p}R_{IIR}}I_{2}-cV-\gamma kTV-hVR_{AIR},\\ \;\frac{dR_{IIR}}{dt}=\psi V-bR_{IIR},\\ \frac{dR_{AIR}}{dt}=fV+\beta R_{AIR}. \end{array}$$

In this model, the IIR (*R*_*IIR*_) represent cytokines and recruited cells of the IIR, e.g., neutrophils and macrophages while the AIR (*R*_*AIR*_) is represented as humoral immune response via B-cells and antibodies. With the free virus, the *R*_*IIR*_ expands at constant rate ψ and dies at constant rate *b*. Herein, the effect of the IIR is modeled by blocking the virus production rate *p*. The *R*_*AIR*_ is triggered by the virus and recruited at constant rate *f*. By clonal expansion at rate constant β, the *R*_*AIR*_ is activated and neutralizes the virus with constant rate *h*. Note that in this coupled model the dead cells *D* are replaced by new target cells at constant rate *r* that represents the regeneration of susceptible cells (Handel et al., 2010).

## Modeling HIV infections

HIV infects cells of the immune system and causes AIDS within 2–15 years post infection. In 2016, the World Health Organization (WHO) estimated that globally 36.7 million people were living with HIV with 1.8 million new infections in 2016. More than 19.5 million of these were treated with a lifelong antiretroviral therapy (ART), the current standard of care. Nowadays, the replication of HIV can be controlled and suppressed by the combination of at least three antiretroviral drugs, e.g., by reverse transcriptase inhibitors and protease inhibitors (World Health Organization, 2017b). These drugs have to be taken live-long and treatment regimens need to be adapted regularly to keep the infection under control. To date, no curative drugs and no vaccine against HIV are available.

### Viral dynamics

**Figure 2:**
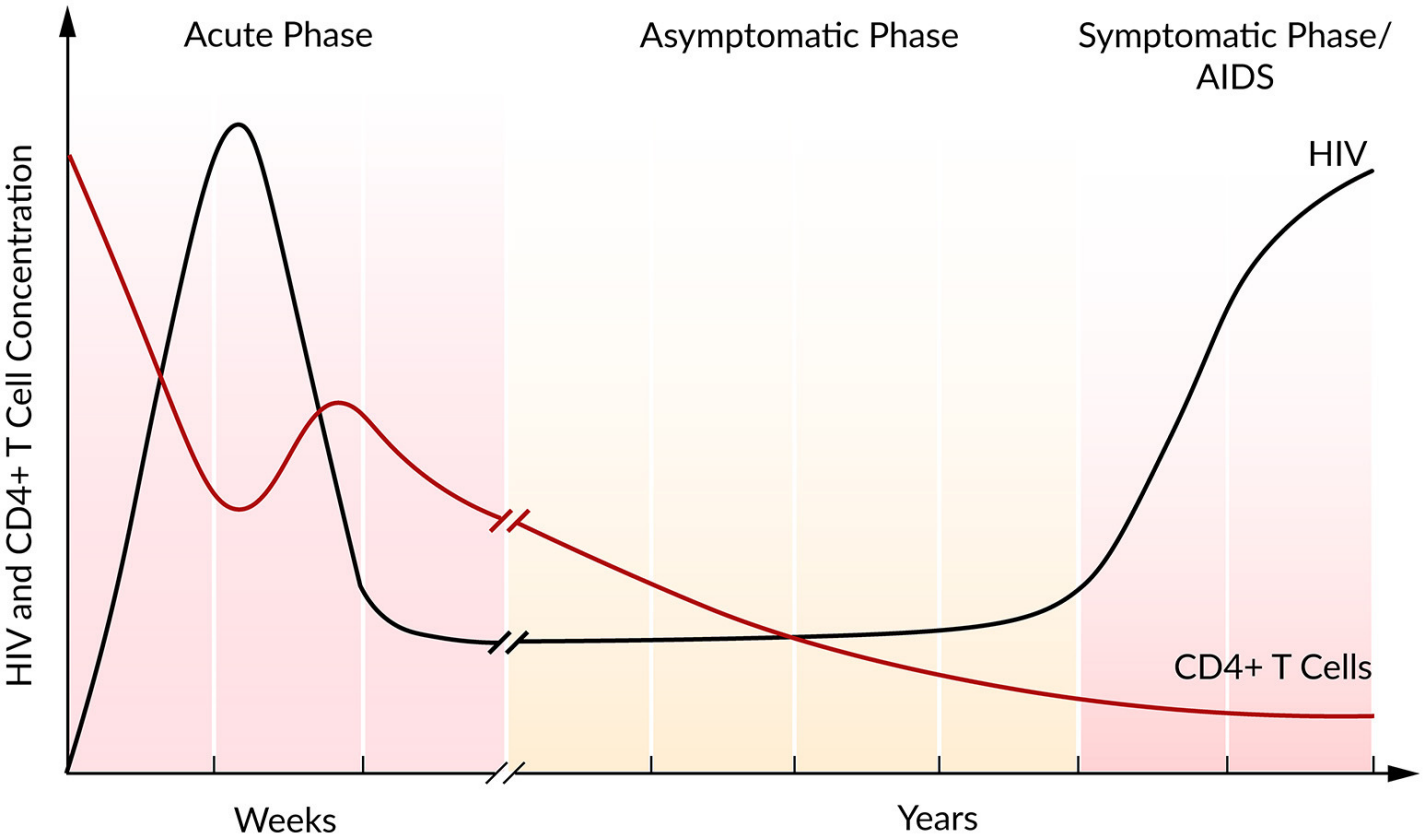
Course of HIV and CD4+ T cell concentration of an untreated HIV infection. Based on Fauci et al. (1996).

In the majority of cases, the infection with HIV follows a typical pattern of three different phases (Figure 2) (Simon and Ho, 2003; Munier and Kelleher, 2007). The first weeks post infection, the acute phase, are characterized by an exponential increase in viral load accompanied by a rapid depletion of CD4+ T cells, the target cells of HIV. Soon after the infection, the immune response kicks in and initiates a decrease in viral load until a constant level, the so-called set point, is reached (Ho, 1996). Within this second asymptomatic phase, the virus persists for years while CD4+ T cells continuously and slowly decline. The third and final phase is characterized by a gradual depletion in CD4+ T cells that is correlated with a strong increase in the viral plasma concentration leading to AIDS (Alizon and Magnus, 2012; Maartens et al., 2014).

During the asymptomatic phase, the viral set point is maintained by a balance in viral clearance and the total virion production rate (*p*_*total*_ = *cV*). Therefore, a strong increase in viral load that is associated with a lower viral clearance rate indicates a stronger total viral production rate *p*_*total*_ > *cV*, while a decrease in viral load refers to a higher clearance rate, *p*_*total*_ < *cV*. Perturbations of this system equilibrium, e.g., by blocking viral production, lead to information on the rate constants and insights into the course of the viral infection and the potential of antiviral interventions (Perelson, 2002). At steady state and in the absence of ART, it has been estimated that HIV is a rapidly replicating virus that produces 10^10^ virions per day. Furthermore, a rapid virus replication also requires strong viral clearance to maintain the equilibrium (Perelson et al., 1996; Ramratnam et al., 1999).

HIV replicates in CD4+ T cells, which are represented by the target and infected cells in the target cell-limited model. With a modified target cell-limited model, Ribeiro et al. (2010) investigated the very early plasma viremia post exposure to HIV in 47 HIV-positive patients. After a time delay of 24 h where the virus became detectable (>50 RNA copies per mL), simulations have shown an initial viral doubling time of 0.65 days. Viral load peaked at 10^6^ HIV RNA copies per mL after 14 days. The subsequent viral decline was characterized by a virion half-life of 1.2 days (*c* = 0.6 day^−1^). Moreover, for this early infection stage, the authors calculated the basic reproductive ratio of *R*_0_ ~ 8, indicating rapid viral spread and the necessity of an early intervention in order to reduce viral spread and to prevent development of chronicity (Ribeiro et al., 2010). By measuring the viral load in 10 HIV-positive patients for on average the first 100 days during primary infection, Stafford et al. (2000) have shown that the target cell-limited model is able to reproduce the interpatient variability within the highly dynamic initial phase post infection. The model simulations provided strong evidence that the initial viral load decline is due to a limitation in target cells with an estimated lifetime of 2.5 days (δ = 0.39 day^−1^) for infected virus-producing cells. However, the target cell-limited model was not able to mimic the data in all the patients equally well. Therefore, the authors suggested that processes not included in the model, such as an involvement of the immune response by CD8+ T cells or destruction of infected cells by cytotoxic T lymphocytes (CTL), might be associated with the stronger than predicted decrease of viral load observed in some patients (Stafford et al., 2000).

### Antiretroviral therapy

**Figure 3:**
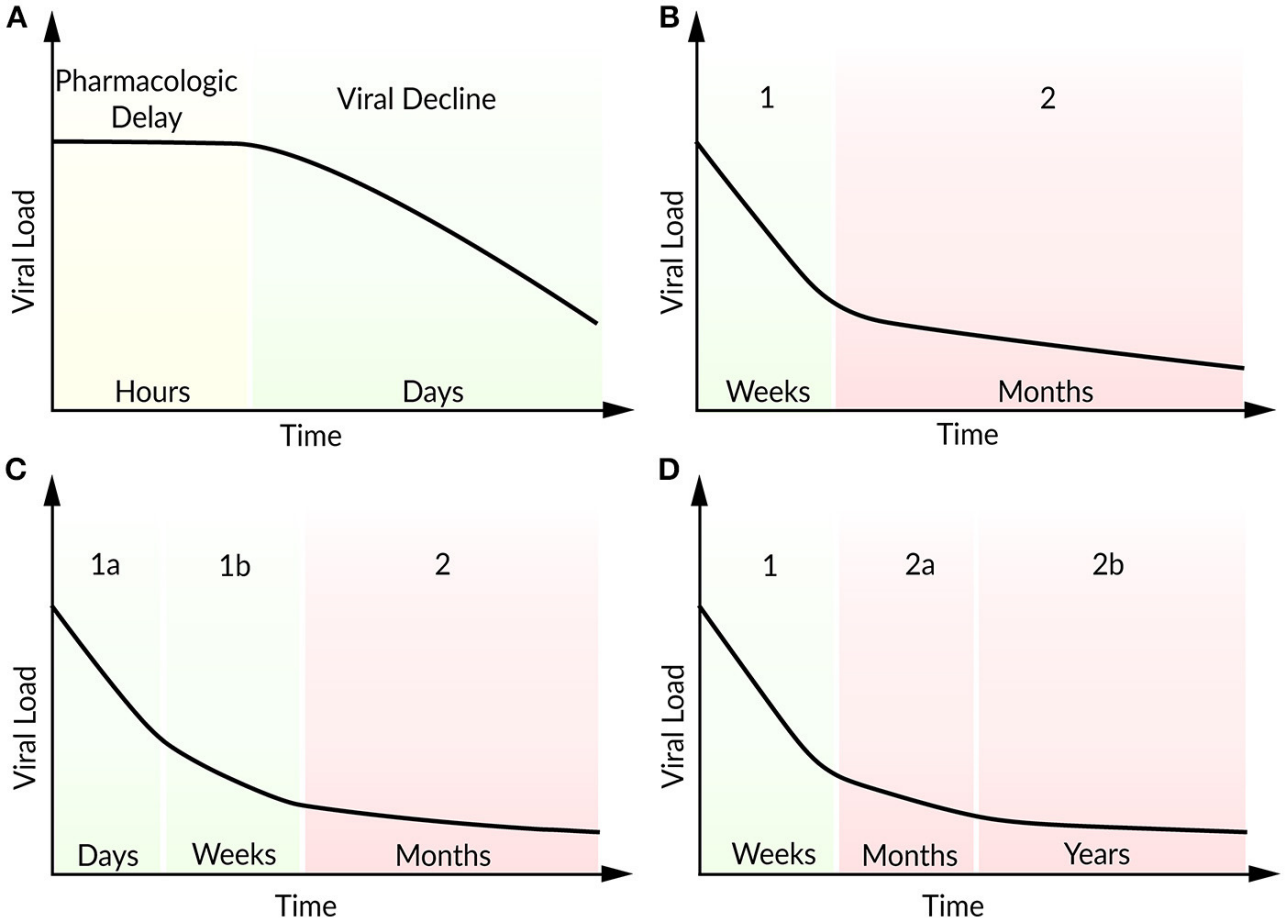
Schematic illustration of viral load decline after onset of ART. **(A)** Viral decline following a pharmacokinetic delay, **(B)** characteristic biphasic decline (phase 1 and 2), **(C)** two sub-phases (1a and 1b) within the first phase, **(D)** two sub-phases (2a and 2b) within the second phase.

For more than 20 years, HIV-positive patients are treated with a combination of antiretroviral drugs. To analyze the effects of an antiviral treatment regimen, the target cell-limited model can be modified to include the effects of reverse transcriptase inhibitors (ε_*k*_) that block viral infectivity (*k*) and protease inhibitors (ε_*p*_) which reduce viral production (*p*) (Neumann, 1998). The effect of a protease inhibitor has been investigated within the first 7 days after the oral administration of Ritonavir (Perelson et al., 1996). Following a pharmacokinetic delay, the patients responded well to the Ritonavir treatment with a continuous decline in plasma viral load. In order to study the viral decline under ART, Perelson et al. (1996) modified the target cell-limited model by the assumption that by the time of drug administration newly produced virions are non-infectious. After a pharmacokinetic delay of about 1.25 days, the model reproduced the strong decline in plasma viremia according to the Ritonavir-treated patients (Figure 3A). The model predicted lifetimes of 2.2 days for virus-producing infected cells and 0.3 days for virions (Perelson et al., 1996). Note that at the onset of ART, the system is assumed to be in steady state. By studying the long-term combination therapy of the protease inhibitor Nelfinavir and the reverse transcriptase inhibitors Zidovudine and Lamivudine, all the patients responded in a similar viral decline pattern (Figure 3B). After initiation of ART, a biphasic viral decline has been observed: a rapid initial reduction in viral load and productively infected cells (phase 1) followed by a slower decrease (phase 2). Perelson et al. (1997) integrated long-lived CD4+ T cells and latently infected lymphocytes that become productively virus-producing cells upon activation as second sources of virus into the target cell-limited model. The authors identified long-lived infected CD4+ T cells with a half-life of 14.1 days (compared to a half-life of 1.1 days of short-lived infected cells) and the continuous release of trapped virus as the main contributors for the second phase (Perelson et al., 1997). Subsequent studies have found more accurate estimates for the virion half-life with 28–110 min in HIV-positive patients under plasma apheresis (Ramratnam et al., 1999) and productively-infected CD4+ T cell half-life of 0.7 days under combination therapy (Markowitz et al., 2003). The continuous viral replication upon activation that is associated with viral persistence represents the challenge in finding a cure for HIV. Even highly active antiretroviral therapy (HAART) does not stop viral production completely, but can achieve a suppression of the viral load in plasma below levels of detection (<50 RNA copies per mL). It is assumed that the main reason for failure to achieve a cure is viral latency. At the same time, the transmission of drug-resistant virus strains is increasing, resulting in increasing treatment failure rates (Little et al., 2002).

In patients with multi-drug resistant virus, Raltegravir represents a promising new antiviral drug that inhibits integrase and hence prevents the strand transfer of proviral DNA into the host-cell genome (Steigbigel et al., 2008). Andrade et al. (2015) analyzed the effect of Raltegravir in monotherapy and in combination with the reverse transcript inhibitors Emtricitabine and Tenofovir Disoproxil Fumarate by an extended target cell-limited model that discriminates between infected cells with and without integrated viral DNA. The authors found a biphasic decline within the first phase during the first 10 days after onset of ART (Figure 3C). A loss in infected cells with integrated viral DNA and a half-life of ~0.8 days (in agreement with 0.7 days in Markowitz et al., 2003) has been identified as the main contributor to the first sub-phase (phase 1a). Cell loss and in addition the integration of provirus into pre-integrated infected cells have been identified as key contributors to the slower decay in the second sub-phase (phase 1b). Interestingly, the half-life of unintegrated infected cells depended strongly on the provirus integration rate and has been estimated to lie between 4 and 7 days (Andrade et al., 2015). Cardozo et al. (2017) generalized the model of Andrade et al. (2015) by taking long-lived infected cells and the effect of protease inhibitor into account in order to investigate the viral decay in presence or absence of Raltegravir therapy (Cardozo et al., 2017). Herein, the therapy containing the integrase strand transfer inhibitor Raltegravir replaced as well the first phase by two sub-phases. The traditional therapy regimen without Raltegravir has shown the typical biphasic decline in viral load. Under Raltegravir therapy, the first phase was associated with the loss of short-lived cells while the second phase corresponded to the loss of long-lived cells with a half-life of ~33 days. The decline of the short-lived cell population within the first phase can be further separated by a loss of productively virus-producing cells with a half-life of ~0.8 days in sub-phase 1a and by pre-integration cells that showed a half-life of ~1.8 days. Furthermore, long-lived cells showed a shorter viral integration rate (0.05 day^−1^) compared to short-lived cells with a viral integration rate of 2.6 day^−1^ (Cardozo et al., 2017).

Moreover, in patients under long-term ART, Palmer et al. (2008) studied a second biphasic decline within the second phase referring to two sources of viremia with persisting virus for more than 7 years (Figure 3D) (Palmer et al., 2008). Kim and Perelson (2006) introduced a model extended by the proliferation of latently infected CD4+ T cells without being activated (bystander proliferation) and explained the persistence of a latent reservoir (Kim and Perelson, 2006). Chomont et al. (2009) observed these results experimentally and identified two different memory T cells contributing to the long-lasting reservoir and thus the persistence of HIV for decades (Chomont et al., 2009). Therefore, an early antiretroviral intervention is necessary to limit the size of the latent reservoir.

However, to understand the effect of ART within the host cell, a comprehensive investigation of the viral life cycle is necessary. Reddy and Yin (1999) described a detailed model of the intracellular viral growth starting with reverse transcription to particle production and maturation. Their simulation results and sensitivity analysis predicted a higher monotherapeutic effect of reverse transcription inhibitors (ε_*k*_) than protease inhibitors (ε_*p*_). A 10-fold decrease in viral reverse transcriptase reduced the overall viral replication to <1%. Moreover, they found that the 10-fold inhibition of Rev—a regulator protein of virion production—increased the viral production, whereas a 100-fold inhibition decreased the production of virions (Reddy and Yin, 1999). These results indicate that incomplete inhibition might be compensated that might lead to adverse and unwanted effects.

As with other RNA viruses, the HIV genome is highly variable, posing its own challenges to treatment. For example, the trans-activating regulatory protein Tat controls gene expression and activates viral transcription by binding at the trans-activating response element TAR (Karn and Stoltzfus, 2012). It has been shown that point mutations in Tat may lead to more virulent HIV strains with higher stability and transcription efficiency which aggravate the development of novel antiretroviral drugs (Ronsard et al., 2014, 2017a; Ronsard, 2017b). On the other hand, Tat might be a promising vaccine candidate and has shown potential in the reduction of HIV plasma viremia associated with a reduced immune activation (Gray et al., 2016). Taking genomic variability and genetic drift of HIV under treatment into account is an important issue, and several authors have modeled the within-host evolution of HIV under selective pressure, see for example (Ribeiro and Bonhoeffer, 2000; Wodarz and Lloyd, 2004; Ball et al., 2007; Rong et al., 2007a,b; Xiao et al., 2013).

### Role of CD8+ T cells and the latent reservoir

Interestingly, within HIV cohort studies [VISCONTI (Goujard et al., 2012; Sáez-Cirión et al., 2013) and SPARTAC (Salgado et al., 2011)] patients have been identified who were able to control HIV infection (<50 RNA copies per mL) after ART cessation, so-called post-treatment controllers. Moreover, there are HIV infected patients (elite controllers) which are able to control and suppress plasma viral load (<50 RNA copies per mL) naturally without ART. In HIV long-term non-progressors, significantly stronger and more complex CD8+ T cell responses associated with higher HIV directed CD8+ proliferation and more effective killing of infected CD4+ T cells have been observed (O'Connell et al., 2009). Recently, Conway and Perelson (2015) extended the target cell-limited model by CTL and latently infected CD4+ cells. Herein, for a very strong immune response, the same dynamics as in elite controllers has been observed. With respect to the size of the latent reservoir, an insufficient CTL response resulted either in viral rebound or post-treatment control. Therefore, post-treatment control after ART cessation depends strongly on a small latent reservoir. The authors suggested therapeutic vaccination to increase the strength of the CTL killing rate and latent reversing agents to decrease the size of the latent reservoir (Conway and Perelson, 2015).

Promising advances in the treatment of latent HIV have been made by an induction and clearing strategy of the latent reservoir, so-called “kick and kill.” Kick refers to the activation of the HIV provirus replication of the latent reservoir, while kill refers to the clearance of reactivated cells by the immune system and/or ART (Barton et al., 2013). For example, vaccinating HIV-positive patients under HAART has shown a transient increase of CD4+ T cell killing and thus a temporary decrease of the latent reservoir (Persaud et al., 2011). Another possibility to activate HIV in latent CD4+ T cells may be achieved by Vorinostat, a histone deacetylase inhibitor. Vorinostat has been shown to be very effective in the induction of HIV transcription in resting memory CD4+ T cells in patients under ART (Archin et al., 2012). To understand the effect of Vorinostat on resting CD4+ cells and the whole latent reservoir, Ke et al. (2015) have developed mathematical models of latency under Vorinostat therapy. They could show that Vorinostat transiently activates HIV transcription but does not reduce the reservoir itself, indicating the necessity of a combination therapy (Ke et al., 2015). In 2015, HIV/AIDS disappeared from the list of the top 10 causes of deaths, indicating that substantial progress has been made by extensively investigating HIV, both experimentally and theoretically. Moreover, from 2000 to 2015 the number of people receiving ART increased from 770,000 to 18.2 million, with a projection of 30 million people on ART in 2020 (Boerma et al., 2015).

## Hepatitis C virus

The blood-borne HCV is a plus-strand RNA virus that causes the acute hepatitis C infection, as well as life-threatening chronic hepatitis C-related diseases like liver cirrhosis or hepatocellular carcinoma. Worldwide, ~80 million people live with chronic hepatitis C with annually 400,000 deaths. For decades, the therapy of choice was based on standard or pegylated interferon (IFN/peg-IFN) and achieved a sustained virologic responses (SVR) between 30 and 60% for IFN and 40–65% for peg-IFN, depending on the HCV genotype and disease progression. Recently, DAAs were introduced to HCV treatment, and increased cure rates to over 90% (World Health Organization, 2016b).

### Viral dynamics

During an acute HCV infection, the viral load increases in a biphasic manner, reaching a peak of 10^5^-10^7^ IU per mL and is then cleared by the host immune response. However, 55–85% of HCV patients develop chronic hepatitis C with persisting virus (Hoofnagle, 2002). Thimme et al. (2001) found that the outcome of an acute infection and its correlation with HCV control is associated with a sustained CD4+ and CD8+ T cell response (Thimme et al., 2001). The biphasic increase in the plasma viral load has been characterized by a rapid viral rise followed by a slower increase, with viral doubling times in the two phases of 0.5 and 7.5 days, respectively (Major et al., 2004). In between these two phases, Dahari et al. (2005) observed a transient reduction in viremia and introduced a generalized model that allows the inhibition of virus production. Model simulations suggest that during that transient decrease of plasma viral load, the endogenous type I IFN response blocks virion production, but without controlling the HCV replication completely (Dahari et al., 2005).

### Antiviral treatment

To estimate the absolute efficacy of IFN therapy, Neumann (1998) integrated the effect of IFN-α into the target cell-limited model by inhibiting the virus production rate (*p*) or the *de novo* infection rate (*k*). After initiation of IFN-α therapy, plasma viral load declined in a similar biphasic manner as has been observed in HIV patients, with a strong first followed by a slower second decrease, resulting in persistence of HCV. Following a pharmacokinetic delay of ~9 h, this biphasic viral decline could be reproduced in the model by partial blocking of the viral production rate with ε_*p*_ < 1. Furthermore, the clearance of free virions (*c*) and therapy efficacy (ε) led to the initial rapid decline while the loss of infected cells (δ) represented the second slower phase. Due to a dose-dependent virus reduction, the authors suggested to increase IFN dosage in treatment for a better antiviral effect early in the infection. They estimated the virion half-life to be ~2.7 h (*c* = 6.2 day^−1^) and the infected cell half-life of 1.7–70 days (δ = 0.14 day^−1^). Before the initiation of therapy, the estimated virion production and clearance rates were 10^12^ virions per day (Neumann, 1998).

In some patients, a triphasic decline with a more rapid third phase has been observed under treatment with pegylated IFN-α in monotherapy or in combination with Ribavirin. Herrmann et al. (2003) suggested the possibility that the third phase decline could be the result of an infected cell loss enhanced by immune-mediated clearance of Ribavirin (Herrmann et al., 2003). In some patients with the triphasic decline, the second phase represented a 4–28 days lasting shoulder phase where HCV was slowly decreasing or remained constant. With a modified model concerning the proliferation of uninfected and infected cells, Dahari et al. (2007b) could reproduce this triphasic pattern only if the majority of hepatocytes were assumed infected. Furthermore, an uninfected hepatocyte proliferation rate higher than the rate of infected cell loss resulted in that almost balanced shoulder phase. According to model simulations, the shoulder phase or even a biphasic viral decline are not observed if Ribavirin effects infected cell loss (δ) or inhibits the viral production rate (ε_*p*_). The authors suggested that the rapidly decreasing third phase in patients with combination therapy of peg-IFN and Ribavirin might be explained by a mutagenic effect (Dahari et al., 2007b).

### Direct acting antivirals

**Figure 4:**
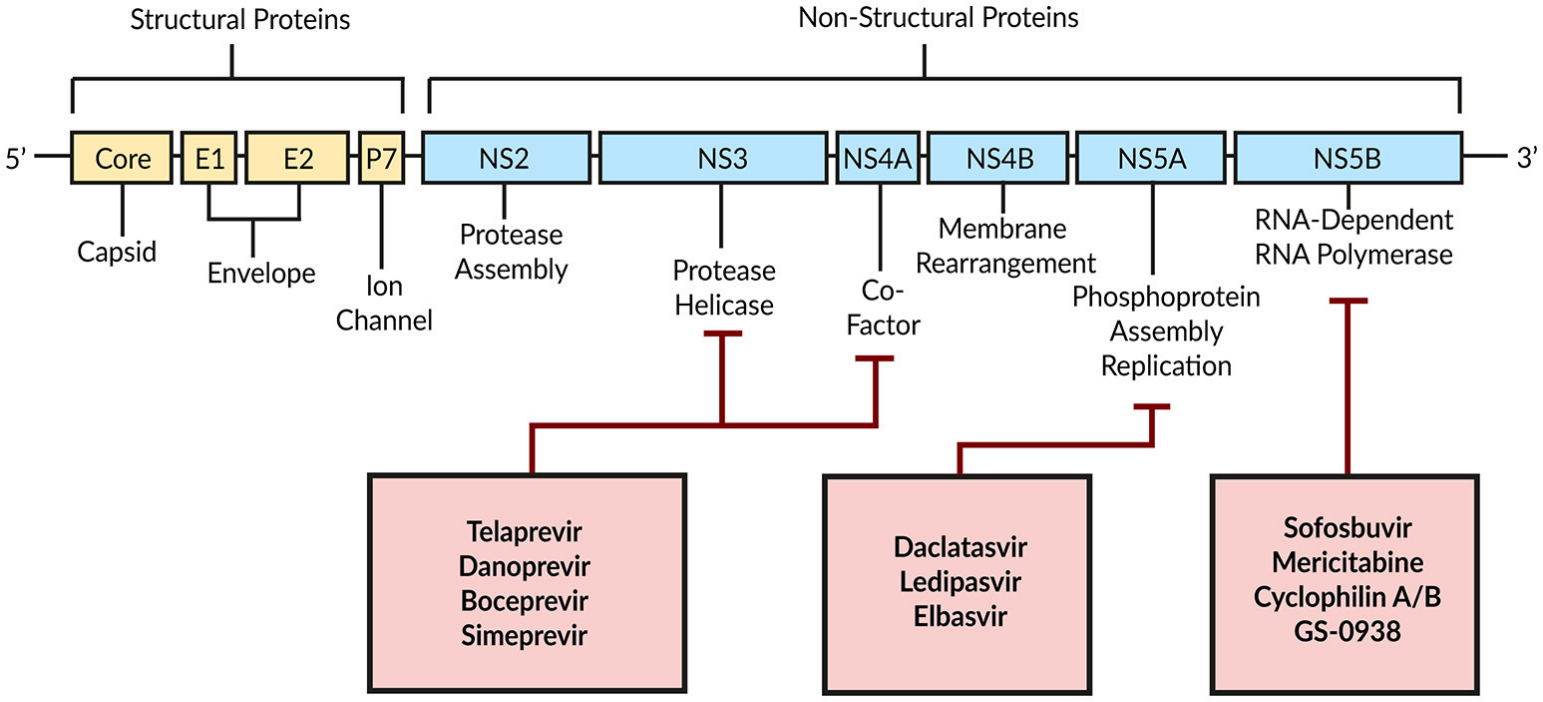
Schematic illustration of DAAs and their HCV target proteins. Based on Bartenschlager et al. (2011).

Combination therapy of peg-IFN with Ribavirin achieves a SVR in only around 50% of patients with HCV genotype 1 (Manns et al., 2001; Fried et al., 2002). With DAAs a new era began by targeting HCV-encoded proteins that are directly involved in the viral life cycle (Figure 4; Scheel and Rice, 2013). A combination of peg-IFN plus Ribavirin with the DAA Telaprevir—an HCV NS3/4A serine protease inhibitor—increased the SVR to around 70% (Jacobson et al., 2011). By modeling the antiviral effect of Telaprevir, Guedj and Perelson (2011) found a 4-fold higher viral decline during the second phase of the biphasic decline with Telaprevir (δ = 0.58 day^−1^) compared to the IFN-based therapy [δ = 0.14 day^−1^; Neumann, 1998]. The authors suggested a higher infected cell death as well as intracellular degradation of viral RNA as modes of action for Telaprevir (Guedj and Perelson, 2011).

### Age-based multi-scale modeling

In 2010, a promising HCV NS5A inhibitor BMS-790052 (Daclatasvir; Kim et al., 2016) has been associated with a 3-log(10) reduction in viremia within the first 24 h, thus offering a highly potent drug (Gao et al., 2010). To understand and compare the mechanisms of action of Daclatasvir and IFN, Guedj et al. (2013) introduced an age-based multi-scale model by integrating intracellular processes, i.e., the antiviral effect on viral RNA replication and particle assembly/secretion, into the target cell-limited model (Equation 4, Figure 2C). For Daclatasvir, the model predicted a 99.0% effective blocking of viral RNA replication (ε_*a*_) and 99.8% effective inhibition of assembly/secretion (ε_*s*_). The viral clearance rate has been estimated as *c* = 22.3 day^−1^, corresponding to an HCV half-life of 45 min, while the intracellular viral RNA had a half-life of on average 11 h. Compared to Daclatasvir, IFN showed a dose-dependent efficacy of 77–96% in blocking intracellular viral replication and only 39% in blocking assembly/secretion, which confirmed the IFN-mediated viral replication inhibition as the main mode of action. Interestingly, the strong antiviral effect of Daclatasvir has been observed only when efficiently blocking both, intracellular viral replication and assembly/secretion. If Daclatasvir was assumed to inhibit only the intracellular viral replication, the kinetics was comparable with that of IFN monotherapy (Guedj et al., 2013). With a similar age-based multi-scale model including intracellular viral RNA replication, viral RNA degradation, and assembly/secretion, Rong et al. (2013) investigated the antiviral effect of the HCV protease inhibitor Danoprevir. They found that Danoprevir was more efficient in inhibiting viral RNA replication (97%) and enhancing viral RNA degradation than inhibiting assembly/secretion (57%). However, for the Danoprevir monotherapy the viral clearance rate has been estimated with *c* = 10.4 day^−1^, corresponding to a virion half-live of 1.6 h (Rong et al., 2013). The age-based multi-scale modeling strategy has shown huge potential in comparing treatment regimens and identifying modes of action of new DAAs.

### IFN-free therapy

Regarding the severe side effects that have been reported with IFN-based therapy (Heim, 2013) and the improved therapeutic response to DAAs, an IFN-free therapy became more and more desirable. Patients treated with the DAA Mericitabine, a nucleoside NS5B HCV polymerase inhibitor, have shown a slower initial viral decline (phase 1) compared to, e.g., the IFN-based therapy, NS5A or non-nucleoside NS5B inhibitors. However, in 40% of the patients, a slow but monophasic viral decline has been observed within the 14 days of Mericitabine treatment. Model predictions have shown that Mericitabine blocks effective viral production whereas the efficacy increases with the accumulation of intracellular phosphates (Guedj et al., 2012). However, a faster initial decline compared to Mericitabine but slower than for other DAAs has been found by evaluating the efficacy of single and co-treatment with the nucleoside HCV NS5B polymerase inhibitors Sofosbuvir and GS-0938. By comparing mono and combination therapy of DAAs of the same family, it was shown that both drugs alone were highly effective and only minor more effective in combination, suggesting an antiviral combination therapy with DAAs of different families (Guedj et al., 2014).

Clinical trials investigating the combination of Sofosbuvir with Ledipasvir (an HCV NS5A inhibitor) with and without Ribavirin have proven highly effective and safe with a SVR >90% (Afdhal et al., 2014a,b; Kowdley et al., 2014). Using a mathematical model, Dahari et al. (2016) analyzed the curing time of Sofosbuvir in combination with either Daclatasvir, Simeprevir, or Ledipasvir within a 12-week treatment duration in 58 patients with chronic hepatitis C. Their simulations show that 98% of patients achieved a SVR with less than one remaining hepatitis C virion. Interestingly, after 6 weeks of treatment, 100% of patients have shown viral loads < 15 IU per mL and no detectable virions in 91% of patients. Additionally, the model predicted that therapy could be shortened in more than 80% of the patients, resulting in a reduce in medication costs by 16–20% (Dahari et al., 2016).

### Host factor targeting and intracellular models

A limitation of the DAA-based therapy is the possibility of developing viral resistance, i.e., emergence of drug-escaping variants dependent on patient groups, HCV genotype, and treatment regimen (Pawlotsky, 2016). In patients treated with Telaprevir over a period of 14-days, Kieffer et al. (2007) found not only an increase in plasma viral load, but also an increase in drug-resistant variants, which replaced the wild-type HCV almost completely at day 15 (Kieffer et al., 2007). Therefore, attention must be paid to finding an effective therapy regimen so that development of drug resistance is avoided. Another alternative treatment strategy is to not directly target the virus, but rather aim for cellular co-factors, since the virus depends strongly on the living host cell for efficient replication. As an example, Cyclophilin B has been identified as a cellular factor modulating the RNA binding activity to HCV NS5B polymerase and thus regulating the HCV replication (Watashi et al., 2005). Liu et al. (2009) reported an interaction of Cyclophilin A and the HCV NS5B polymerase, and predicted that Cyclophilin A as a major key host factor for an active replicase (Liu et al., 2009). Cyclophilin inhibitors such as Alisporivir (Gallay and Lin, 2013), SCY-635 (Hopkins et al., 2012), and NIM 88 (Lawitz et al., 2011) have confirmed the potential in disrupting the HCV replication. This and other findings on host factors have proven how important a detailed understanding of the HCV life cycle and the host interaction is.

To characterize the intracellular viral replication in more detail, Dahari et al. (2007c) developed a detailed mathematical model investigating the single steps of intracellular RNA replication. The model with cytoplasmic translation and RNA replication within a replication compartment has shown that HCV regulates the plus-strand to the minus-strand relation by a strand-specific affinity of HCV NS5B polymerase. Additionally, the authors have shown that the virus benefits from encapsulating its genome replication inside membranous replication sites (Dahari et al., 2007c). Using an extended model and based on detailed measurements of the initial replication kinetics, Binder et al. (2013) mimicked the highly dynamic initial phase within the first hours post infection until steady state of minus-strand RNA, plus-strand RNA, and protein activity. An important finding of this model is the role of the protective replication compartment in which HCV replicates its genome. On the one hand, this compartment appears to protect the virus from antiviral mechanisms and is required for the establishment of a successful replication, on the other hand, this compartment also seems to limit viral growth and thus exerts tight control over the viral dynamics. By the integration of host factors into the model, the authors showed that cellular co-factors that are involved in the formation of the membranous replication sites and the initiation of minus-strand synthesis are responsible for differences in replication efficacy in different cell lines (Binder et al., 2013).

Recently, Benzine et al. (2017) have estimated the half-lives of the replicase complex (a complex of viral and cellular proteins associated with viral genome synthesis) in slowly and rapidly replicating HCV strains. Their mathematical model distinguishes between different viral plus-strand RNA genomes—RNA associated with translation, RNA responsible for RNA synthesis in the membranous web and the replicase complex, as well as RNA that is assembled and packed into virions. The authors estimated replicase complex half-lives of 3.5 h for the fast replicating strain and 9.9 h for the slow replicating strain and speculated that differences in the amino-acids in non-structural (NS) proteins that are responsible for replicase complex formation as well as the interactions with each other or host proteins are underlying the observed differences in half-lives. Furthermore, the antiviral efficacy has been integrated by the effect of the NS5A inhibitor Elbasvir, the NS5B inhibitor Sofosbuvir, and Compound 23. Sofosbuvir inhibits the plus- and minus-strand synthesis, Elbasvir blocks the formation of new replicase complexes and the viral assembly while Compound 23 inhibits the formation of replicase complexes. For the slowly replicating strains, the model predicted that by blocking viral assembly, the RNA is increasingly used for translation while that redirection was very low in fast replicating viral strains (Benzine et al., 2017).

Clausznitzer et al. (2015) developed a multi-scale model combining the target cell-limited model with detailed intracellular replication to investigate the specific effect of Daclatasvir that targets HCV NS5A within the first 2 days post drug administration. For Daclatasvir, the exact mode of action is still unknown. The authors compared different putative mechanisms concerning the initial and long-term dynamics. Blocking viral replication affected the long-term dynamics, while blocking viral assembly/secretion had an effect on the initial and the long-term dynamics. Interestingly, a complete inhibition of viral assembly/secretion did not eradicate the virus. Additionally, it has been shown that the host factor affected the long-term dynamics and represented the main parameter in individual differences in the viral replication efficacy (Clausznitzer et al., 2015).

In a mouse model, Mailly et al. (2015) have shown that the inhibition of Claudin1-mediated viral entry by Claudin1-specific monoclonal antibodies has shown highly effective in preventing HCV infection without the emergence of resistance. By using the target cell-limited model that has been extended by the effect of monoclonal antibodies which inhibit the *de novo* infection rate (*k*), the model predicted the clearance of infected cells and the prevention of new infection (Mailly et al., 2015). Thus, the inhibition of cellular co-factors that mediate viral entry might be a promising strategy to prevent and eradicate HCV.

## Influenza virus

The seasonal influenza is an acute infection of the respiratory tract caused by influenza virus of types A, B, and C. Annually, on average 3–5 million people worldwide are infected. The disease is often associated with severe symptoms and leads to 250,000–500,000 deaths per year. Two classes of antiviral drugs are available against influenza: neuraminidase inhibitors and M2 proton channel blockers. However, the most effective strategy against a seasonal influenza infection is the prevention by a vaccination, which has been proven to be safe and effective for more than 60 years (World Health Organization, 2017c).

### Viral dynamics and immune response

**Figure 5:**
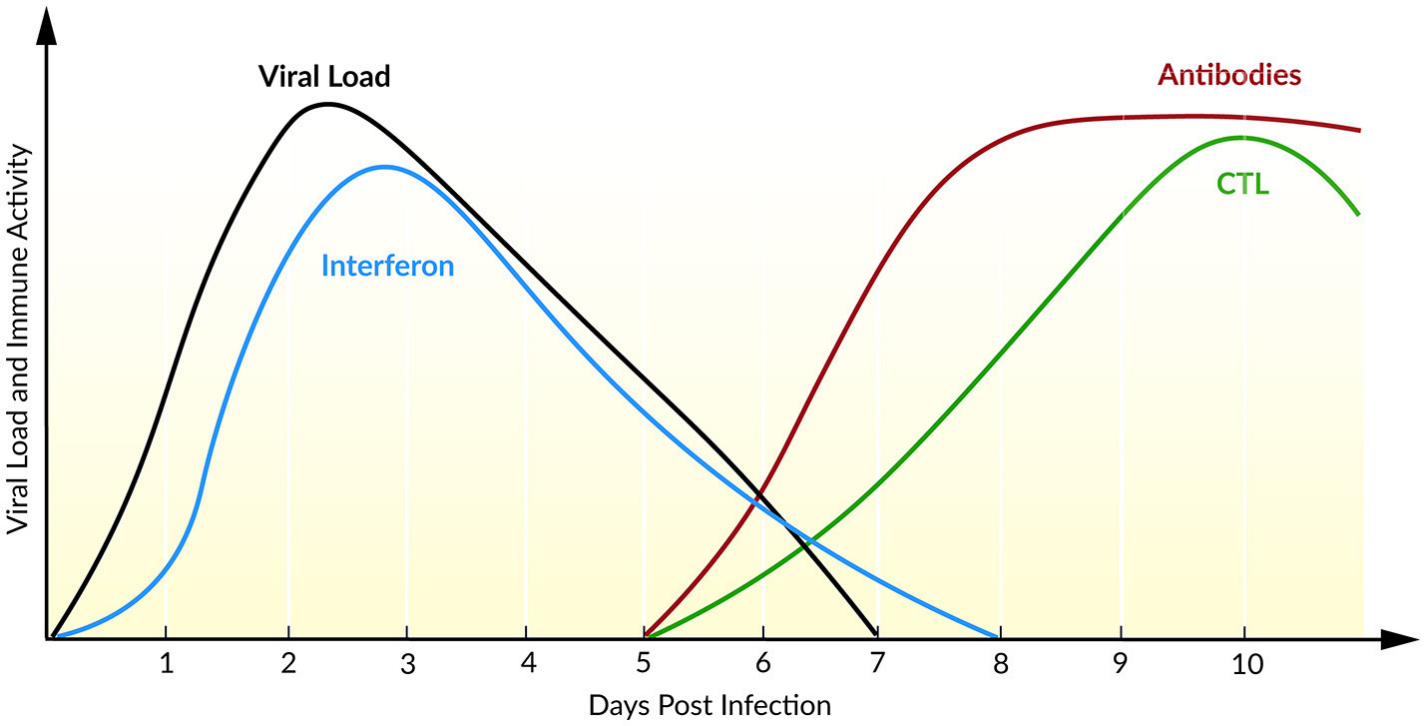
Course of an IAV infection (viral load), the innate immune response (interferon), and the adaptive immune response (antibodies and CTL). Inspired by Beauchemin and Handel (2011) and Wright et al. (2013).

The course of infection with IAV is characterized by an exponential growth of viral load, reaching its maximum 2 days post infection (Figure 5). Within the following days, the viral load declines until the virus becomes undetectable within 6–8 days post infection (Wright et al., 2013). Baccam et al. (2006) modified the target cell-limited model, taking the rapid dynamics of IAV into account. Their model neglects the regeneration and death of target cells (Baccam et al., 2006). With the assumption that progeny virus is undetectable within the first 6–8 h (Sedmak and Grossberg, 1973), an eclipse phase was incorporated into the model that characterized the time delay from cell infection to virus production. In order to model the eclipse phase, the authors introduced two different infected cell populations: not yet virus producing infected cells that are in the eclipse phase (*I*_1_) and actively virus producing infected cells (*I*_2_, Equation 5). With data of patients experimentally infected with IAV, mathematical models with and without the eclipse phase have been analyzed. The authors could show that both models fit the patient data equally well, whereas the eclipse phase model estimated biologically more reasonable parameters with a half-life of free virion of 3.2 h. Furthermore, after a 6 h delay, the infected cells are producing virus for about 5 h, leading to an average lifetime of about 11 h for infected cells. Additionally, the authors calculated the basic reproductive ratio *R*_0_ ~ 22 indicating a rapid viral spread (*R*_0_ ≫ 1) where 1 cell infects ~22 other epithelial cells in the upper respiratory tract, suggesting that an early initiation of treatment is crucial. Interestingly, in 50% of the patients a second peak in viral load has been observed. By extending the target cell-limited model by the effect of IFN (Equation 5), the second peak might be explained by a decreasing antiviral effect of IFN (Baccam et al., 2006).

During IAV infection, IFN is detectable 24 h post infection reaching a maximum after 72–96 h (Roberts et al., 1979). IFN plays a major role in the inhibition of viral infection and establishing an antiviral state (Samuel, 2001). In turn, the IAV protein NS1 has been identified as an IFN antagonist that circumvent the IFN-mediated antiviral response and correlates with pathogenicity (Garcia-Sastre et al., 1998). Saenz et al. (2010) extended the target cell-limited model by the regulation of the IIR. Herein, IFN is released by infected cells which induce an antiviral state by turning target cells into refractory cells. Model predictions demonstrated the major role of IFN in controlling early infection by protecting target cells (Saenz et al., 2010).

To capture the interaction of IAV with the IIR and AIR, Pawelek et al. (2012) included an antiviral state by refractory cells, as well as an IFN-induced infected cell killing into the target cell-limited model. The authors have shown that the early viral infection might be controlled by target cell depletion. The rapid viral post-peak decline could be explained by the enhanced infected cell killing mediated by cytokines, natural killer cells, or other cells activated by IFN. Moreover, the authors were able to mimic the bimodal pattern with a rebound of plasma viral load observed in 50% of the patients (Baccam et al., 2006). They assume that this second peak is due to a loss of the antiviral effect of IFN leading to a recovery of target cells (Pawelek et al., 2012). By comparing the dynamics of four different IAV strains in a mouse model, Manchanda et al. (2014) have shown a strain-specific rebound in viremia leading to a second peak. Furthermore, model predictions explained the rebound by persistent inflammation that correlated with disease severity (Manchanda et al., 2014).

The AIR is mainly mediated by CTLs and antibodies which appear at day 5 after primary infection and at day 3 after reinfection, resulting in a faster memory cell-mediated secondary response (Tamura and Kurata, 2004). Handel et al. (2010) extended the target cell-limited model by simple defense mechanisms of immune mediators, e.g., inflammatory cytokines, as well as antibodies or CTLs (Equation 6). It has been shown that the models with either antibody (killing of free virions) or the CTL-mediated immune response (killing of infected cells) fit the data equally well. A distinction of the underlying mechanisms of the AIR was not possible with the available data (Handel et al., 2010). Miao et al. (2010) combined CTL and antibodies, IgG and IgM, within a mathematical model and confirmed the necessity of CTL and IgM in infection clearance, leading to average half-lives for infected cells of ~0.5 days and for free virions of ~1.8 min. In the absence of an AIR (days 0–5), the half-lives for infected cells have been estimated with ~1.2 days and for free virions ~4 h. Furthermore, the model predicted the contribution of CTLs in killing infected cells while mainly IgM cleared the viral load. Due to a low contribution of IgG in primary infection clearance, the authors suggested a role of IgG together with CD4+ T cells in generating a memory and therefore a second immune response (Miao et al., 2010).

### Risk factor age

The recommended prevention of an influenza infection is a vaccination that reduces severity, complications, and deaths especially in elderly. However, due to a lower antibody response in elderly (age >65 years) the vaccine efficacy is only 17–53% compared with 70–90% in young adults (Goodwin et al., 2006). Hernandez-Vargas et al. (2014) studied the impact of age on the immune response to the course of IAV infection and have shown a limited stimulation of the adaptive immune cells that led to a reduced viral growth with a 1.5 lower *R*_0_ in immune naïve aged mice. Additionally, a delayed (1–2 days) infection clearance correlated with a delayed increase of CD8+ T cells in aged mice, indicating a key role of CD8+ T cells in infection clearance. Therefore, the 10-fold lower viral burden might trigger the immune response insufficiently, explaining the striking difference between infection control and viral titers in elderly and young mice (Hernandez-Vargas et al., 2014). However, these experimental results and modeling predictions are valid for immune naïve aged mice. To study the efficacy of vaccination in elderly, the validation of these results in humans would be appropriate, but is obviously more complicated.

Modeling the effect of CD8+ T cell populations to recurrent IAV infections, Zarnitsyna et al. (2016) have shown that an increase in CD8+ T cell levels led to a decreased viral load and a shorter recovery time. The model of Cao et al. (2016) confirmed the relationship of a faster recovery with an increased level of effector CD8+ T cells. Thus, the induction of CD8+ T cells might be a promising vaccination strategy instead of boosting the antibody response that might lead to antigenic mutations and constantly evolving new influenza strains (Cao et al., 2016; Zarnitsyna et al., 2016).

### Antiviral drugs

The effect of Amantadine, an antiviral agent acting as an M2 ion channel blocker, has been included into the eclipse model (Baccam et al., 2006) by affecting the infection rate (*k*) of target cells by virions. The authors show that the maximum drug efficacy for Amantadine is only 74%, this can be explained by a possible rapid development of drug resistance. For the characterization of the viral dynamics under Adamantane treatment (e.g., Amantadine), it is therefore important to take the emergence of drug-resistance into account (Beauchemin et al., 2008).

Canini et al. (2014) investigated the effect of Oseltamivir (a neuraminidase inhibitor) using a model combining antiviral treatment regimen, IIR, and AIR, as well as a scoring system for symptoms, and the emergence of drug resistance as a random event. The authors show that the prophylactic use (pre-symptomatic phase) of Oseltamivir in low doses may cause a 27% higher emergence of drug resistance during the incubation period, due to an insufficient AIR, e.g., by natural killer cells. The initiation and duration of treatment, drug doses, as well as treatment frequency have been identified as crucial factors for the emergence of drug resistance (Canini et al., 2014). Kamal et al. (2015) studied the time course of influenza infection with and without Oseltamivir that had an effect on the virion production rate by inhibiting the release of newly produced virions (viral shedding). They have shown that a sooner initiation of Oseltamivir treatment correlates with a decreased viral secretion duration. By investigating the effect of a combined treatment, they found that the effect of Oseltamivir together with an antiviral drug affecting viral clearance had significant better effects reducing viral load, regardless of the onset of therapy (Kamal et al., 2015).

Heldt et al. (2013) developed an age-based multi-scale model combining the viral life cycle with cell-to-cell transmission with the aim to investigate the effect of DAAs. The authors found the most promising antiviral strategy by interfering with viral transcription, replication, protein synthesis, nuclear export, and assembly/secretion, while inhibiting early steps in replication—virus entry—caused only a delayed virus production. They additionally showed that some drugs could in fact increase the virus production, indicating how important a detailed understanding of the dynamic events in the virus life cycle is (Heldt et al., 2013). Schelker et al. (2016) investigated early events in the viral life cycle within a 3D diffusion modeling approach that identified the time point of endocytosis and the distance of diffusion to the nucleus as a bottleneck, supporting cytosolic degradation as limiting factors for efficient virus replication (Schelker et al., 2016).

## Other viruses

### Ebola virus

From 2013 on, EBOV of the type Zaire has caused the largest outbreak to date in West Africa with reported 29,000 disease cases and 11,000 deaths. An untreated acute Ebola infection causes severe illness with a fatality rate of on average 50% (World Health Organization, 2017a). EBOV is a negative-stranded RNA virus that replicates in immune cells, with the ability to persist in immune-privileged sites such as the central nervous system and may thus lead to viral relapse (Jacobs et al., 2016). No specific treatment is currently available, but recently a clinical trial with a newly developed vaccine (rVSV-ZEBOV) has shown to be highly protective against the Ebola disease (Henao-Restrepo et al., 2017).

To capture the Ebola infection dynamics, Nguyen et al. (2015) used the target cell-limited model and compared EBOV to pandemic IAV. EBOV infection time is significantly slower than IAV infection time (9.5 h vs. 30–80 min) (Holder et al., 2011; Pinilla et al., 2012; Nguyen et al., 2015). Furthermore, the viral replication rate has been estimated as ~63 ffu/mL day^−1^ cell^−1^, EBOV is hence highly efficient with a virion half-live of ~23 h (*c* = 1.05 day^−1^) (Nguyen et al., 2015). Unfortunately, these results are uncertain due to parameter identifiability problems. Nonetheless, the target cell-limited model confirmed the viral growth seen in experimental data, starting at day 3 post infection with a complete target cell depletion at day 6. Madelain et al. (2015) extended the target cell-limited model by an eclipse phase (non-/virus-producing infected cells) and found a half-life for virus-producing infected cells of 6.4 h and a basic reproductive ratio of *R*_0_ ~ 9. The authors furthermore studied the antiviral effect in mice treated with Favipiravir, an antiviral drug that blocks the RNA-dependent RNA polymerase in a broad spectrum of RNA viruses (Furuta et al., 2013). By inhibiting the virus production rate *p*, they found a sharp decrease in viral load that was associated with an increasing drug efficacy of 95, 98.5, and 99.6% at days 2, 3, and 6 after the onset of treatment. Since Favipiravir achieves its maximal efficacy after 3 days, an early treatment initiation is suggested (Madelain et al., 2015). With patient data of survivors and fatalities from the Uganda Ebola disease outbreak in 2000/2001, Martyushev et al. (2016) studied the relationship between virus replication and disease severity. For this purpose, they extended the target cell-limited model by two target cell populations: potential target cells (*T*_2_), that are recruited via proinflammatory cytokines (e.g., recruited macrophages, hepatocytes, splenocytes, and endotheliocytes), which become susceptible target cells (*T*_1_), that are the primary target for viral replication (e.g., macrophages and dendritic cells). Ebola disease severity is described by a 2 log(10) higher plasma viral load, that is correlated with an extensive recruitment of potential target cells and a 2.2-fold higher basic reproductive ratio; *R*_0_ ~ 6 for fatal cases and *R*_0_ ~ 2.8 for nonfatal cases. Hence, the higher viral load in fatal cases and a massive infection/hypersecretion of cytokines by active virus-producing replication cells is associated with the potential severity of the Ebola disease (Wauquier et al., 2010; Martyushev et al., 2016). Additionally, antiviral intervention of (i) an antibody-based therapy that affects the *de novo* infection (*k*), (ii) a siRNA-based treatment that blocks viral production (*p*), and (iii) a nucleoside analog-based therapy (e.g., Favipiravir) have been evaluated in mono- and combination therapy. The combination of nucleoside analog-based therapy and siRNA-based turned out to be most efficient if initiated 4 days post symptom onset, while the antibody-based therapy seemed insufficient (Martyushev et al., 2016). The authors then demonstrated that a critical inhibition rate of 80.5% in fatal cases and 58.5% in nonfatal cases is needed to prevent fatal outcomes of the Ebola virus disease.

### Dengue virus

The DENV is a positive-stranded RNA virus, infecting annually 390 million people worldwide. DENV is spread mainly by the mosquitos *Aedes aegypty* and *Aedes albopictus*, which also transmit Chikungunya Virus, Yellow Fever Virus, and ZIKV. There are four serotypes of DENV, causing flu-like illness occasionally associated with severe complications like hemorrhagic fever. A cleared dengue infection provides a serotype-specific lifelong immunization, while secondary infections with another serotype can result in severe dengue disease. Currently, there is no antiviral treatment available, but a recently developed dengue vaccine (CYD-TDV; Villar et al., 2015) is suggested for endemic regions (World Health Organization, 2016a).

To explain inter-individual differences in DENV infection dynamics, Clapham et al. (2014) extended the target cell-limited model by a simple AIR. Moreover, differences between primary and secondary infection could be explained by the variations in the immune response. For a secondary infection, the immune response-related parameters have shown higher values, e.g., the immune cell proliferation rate and the virus clearance rate. Interestingly, the infectivity rate constant (*k*) has also reached higher values in a secondary infection compared to a primary infection, supporting the hypothesis of antibody-dependent enhancement where antibodies mediate virus entry and thus increase the viral infectivity in a secondary infection (Clapham et al., 2014). In a subsequent study, Clapham et al. (2016) investigated the antibody dynamics within a target cell-limited model predicting the role of IgM and IgG in the course of a dengue infection. They showed that a primary infection was mainly cleared by IgM while a secondary infection was cleared by IgG and IgM. These results refer to the key role of IgM in DENV infection clearance. Furthermore, best fitting results have been found by assuming that antibodies directly neutralize free virus compared to a clearance of infected cells, e.g., via antibody-dependent cell cytotoxicity. However, model predictions have shown a short life-span of infected cells with 0.3 days referring to additional immune-mediated clearance mechanisms (Clapham et al., 2016).

Ben-Shachar and Koelle (2014) developed a series of within-host dengue models integrating key players of the IIR and AIR in order to investigate the viral dynamics and development of severe dengue disease. They extended the target cell-limited model only by the IIR and were able to reproduce the viral dynamics in primary infection. Furthermore, they showed that higher rate constants for infectivity (*k*; evidence for antibody-dependent enhancement) and infected cell death (δ; evidence for T cell response with increasing severity) were necessary to mimic the viral dynamics of a secondary infection (Ben-Shachar and Koelle, 2014). Recently, Ben-Shachar et al. (2016) refined these results by investigating serotype-specific differences. The higher infectivity rate constants (*k*) estimated for DENV-2 and DENV-3 compared to DENV-1 in their model were consistent with varying replication efficacy of different dengue serotypes (Ben-Shachar et al., 2016).

With a population-based delay model coupled to the IIR, Schmid et al. (2015) studied the attenuated viral spread of a DENV mutant that is proposed as a vaccine candidate. In their work, they show that the DENV mutant has a faster IFN activation and production which establishes an antiviral state in infected cells and leads to an 8-fold decreased viral production and spread compared to the wildtype DENV. Furthermore, their model shows a stronger impact of the autocrine IFN in comparison to the paracrine effect on reducing viral spread (Schmid et al., 2015).

### Zika virus

ZIKV is a plus-stranded RNA virus that is mainly carried and transmitted by *Aedes* mosquitos, but sexual transmission has as well been reported (Foy et al., 2011; Musso et al., 2015; D'Ortenzio et al., 2016). Human infections with ZIKV usually cause only mild disease with similar symptoms as seen in DENV infections. However, during the recent outbreak in Brazil with estimated 440,000–1,300,000 Zika cases (Heukelbach et al., 2016), ZIKV has been associated with neurologic complications such as Guillain-Barré syndrome and fetal microcephaly (World Health Organization, 2017d).

Recently, Best et al. (2017) developed a series of models with and without incorporation of the immune response and fitted those to plasma viral load data of ZIKV-infected nonhuman primates. Within that model series, the target cell-limited model only extended by an eclipse phase that distinguishes between non-actively and actively virus-producing infected cells was the best-suited model to reproduce the data. Furthermore, the incorporation of key players of the IIR or AIR, e.g., by IFN or natural killer cells, respectively, did not improve the model fitting and thus has been neglected. The simple eclipse phase model estimated an eclipse phase of ~4 h (already observed via modeling in Osuna et al., 2016) and a basic reproductive ratio of *R*_0_ ~ 10.7. The degradation rate of productively infected cells was estimated with δ = 4.5 day^−1^, corresponding to a lifetime of ~5 h. The authors furthermore included the effect of antiviral therapy by inhibition of the viral production rate. With the broad spectrum RNA polymerase inhibitor Favipiravir, the time to undetectable plasma viremia could be reduced by 2 days if the initiation of therapy starts at the time point of infection (*t* = 0 days post infection). The therapy initiation at day 2 post infection led to the same result compared to no drug treatment, leading to undetectable plasma viral load after 5 days post infection (Best et al., 2017). By integrating the immune response via IFN and neutralizing antibodies into the eclipse phase model, Aid et al. (2017) found a positive effect of both in controlling the viral infection in the periphery. The overall best fit was achieved by initiating IFN response at day 1.5 while the activity of neutralizing antibodies started at day 6 (Aid et al., 2017).

## Conclusion

For more than 20 years, the population-based target cell-limited model has been used to describe the dynamics of a variety of viruses. The interdisciplinary research combining experimental measurements and mathematical modeling improved our understanding of virus-host interactions and helped to quantify key parameters of the viral life cycle. Simple mathematical models allowed the investigation of the circumstances that lead to viral eradication or the development of chronic infections with an equilibrium of virus production and immune-mediated clearance. Studying antiviral drug treatments with the target cell-limited model enabled the identification of drug efficacy and modes of action. Moreover, simple extensions of the model led to insights into the different patterns of viral decline during drug treatment and the evaluation of different treatment regimens. By taking the immune system into account, mathematical modeling helped to identify the key players for viral clearance.

A comprehensive and quantitative, dynamic understanding of virus-host interactions is vital for advances in antiviral therapy, and can be achieved by modeling the entire viral life cycle from virus entry to particle production. This would support not only the prediction of more precise modes of action of DAAs, it would also help to identify and evaluate new treatment opportunities or the potential of broad-spectrum antiviral drugs. Drugs that interact directly with viral proteins have shown enormous potential, but may lead to the emergence of virus strain mutations, multi-drug resistance, and treatment failure. Therefore, future research might focus more on resistance free antiviral drugs, e.g., by targeting host factors or by the prevention of viral diseases with vaccination. To support knowledge-based design of such drugs and vaccines, a more comprehensive view of the immune response to viral infections is necessary. Regarding the complex interplay of the first line of defense by the IIR and the establishment of an immune response memory by the AIR, questions arise how the virus hides and circumvents the immune response or why some patients are able to clear an infection that would develop to chronic infection in the majority of patients.

Furthermore, modeling techniques may consider not only the time-dependent dynamics but focus as well more on the spatial scale. By combining time and space scales, agent-based models may help to characterize viral spread in tissue, within organs or in the whole human body. Additionally, the complex interplay between the virus and the immune system may be studied by agent-based models with relatively simple rules (Bauer et al., 2009; Graw and Perelson, 2015; Kumberger et al., 2016). Mathematical modeling addressed important questions concerning the virus-host interactions and may contribute to answering open questions.

## Author contributions

All authors listed have made a substantial, direct and intellectual contribution to the work, and approved it for publication.

### Conflict of interest statement

The authors declare that the research was conducted in the absence of any commercial or financial relationships that could be construed as a potential conflict of interest.

## Abbreviations

AIR: Adaptive Immune Response
ART: Antiretroviral Therapy
CTL: Cytotoxic T lymphocytes
DAA: Direct-Acting Antiviral
DENV: Dengue Virus
EBOV: Ebola Virus
HAART: Highly Active Antiretroviral Therapy
HCV: Hepatitis C Virus
HIV: Human Immunodeficiency Virus
IAV: Influenza A Virus
IFN: Interferon
IIR: Innate Immune Response
NS: Nonstructural Protein
ODE: Ordinary Differential Equation
SVR: Sustained Virologic Response
WHO: World Health Organization
ZIKV: Zika Virus.
